# Vertically oriented mesoporous silica film modified fluorine-doped tin oxide electrode for enhanced electrochemiluminescence detection of lidocaine in serum†

**DOI:** 10.1039/d1ra06375h

**Published:** 2021-10-26

**Authors:** Renchuan Liang, Jinghang Jiang, Yanyan Zheng, Ajabkhan Sailjoi, Jie Chen, Jiyang Liu, Hongxue Li

**Affiliations:** Guangxi Medical University Cancer Hospital, Guangxi Medical University 71 Hedi Road Nanning 530021 PR China lihongxue1616@163.com; Department of Chemistry, Zhejiang Sci-Tech University 928 Second Avenue, Xiasha Higher Education Zone Hangzhou 310018 PR China liujy@zstu.edu.cn

## Abstract

Owing to a nanochannel-based enrichment effect and anti-fouling ability, highly ordered and vertically oriented mesoporous silica thin film (VMSF) modified electrodes have demonstrated their great potential in direct and highly sensitive analysis of complex samples. In this work, a VMSF modified fluorine-doped tin oxide (FTO) electrode (VMSF/FTO) is fabricated for enhanced electrochemiluminescence (ECL) analysis of lidocaine in serum. VMSF with good integrity and mechanical stability can be rapidly and conveniently grown on FTO in a few seconds at room temperature using an electrochemically assisted self-assembly (EASA) method. Due to the strong electrostatic attraction between the cationic ECL probe and negatively charged nanochannel, the VMSF/FTO electrode shows significant enrichment of tris(2,2-bipyridine) ruthenium(ii) (Ru(bpy)_3_^2+^), leading to ∼10 times enhancement of its ECL signal in comparison to the bare FTO electrode. Lidocaine, an anesthetic and antiarrhythmic drug, can act as the co-reactant of Ru(bpy)_3_^2+^ and promote its ECL signal. Sensitive ECL detection of lidocaine is achieved by the sensor in a wide linear range from 10 nM to 50 μM with a low limit-of-detection (LOD) of 8 nM. Combined with the antifouling ability of VMSF, the VMSF/FTO electrode also realizes the accurate and rapid analysis of lidocaine in real serum samples.

## Abstract 2

Sensitive detection of lidocaine in serum is realized using enhanced electrochemiluminescence based on a vertically oriented mesoporous silica film modified fluorine-doped tin oxide electrode.

## Graphical abstract

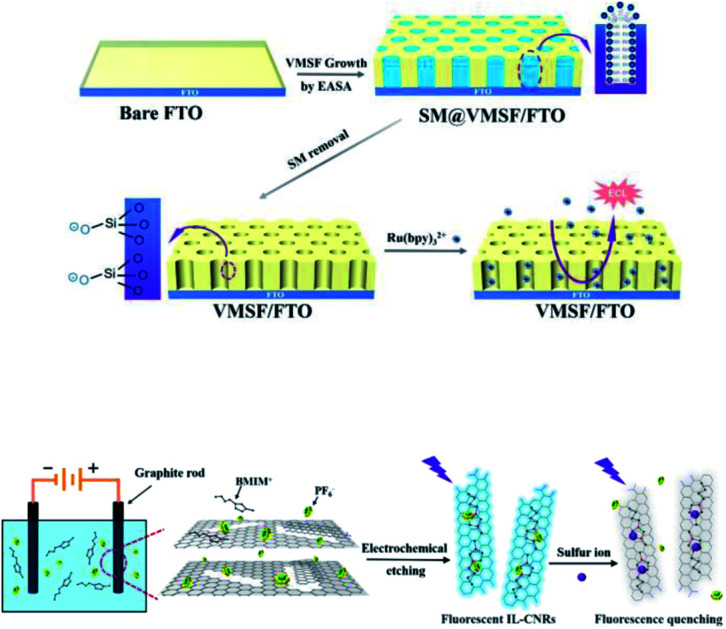

## 1. Introduction

Highly ordered solid-state nanofilms have recently demonstrated great potential in (bio)sensing, molecular separation, energy conversion, catalysis, delivery, and nanofluids owing to their unique advantages of adjustable nanopores, high specific surface area, controlled molecular transportation, and easy integration ability.^1–3^ Amongst such films, the vertically oriented mesoporous silica film (VMSF) is attractive as a mesoporous nanofilm prepared using self-assembled surfactant micelles as templates.^4–6^ VMSF consists of nanochannel array perpendicular to the supporting electrode, which has uniform channel size (generally 2 ∼ 3 nm in diameter) and high density (up to 75 000 per μm^2^).^7–10^ The high density of nanochannel and nanosized thickness ensure efficient diffusion and mass transfer from solution to the supporting electrode. More importantly, VMSF nanochannel has permselectivity based on size, charge and hydrophobicity of molecules because its ultrasmall aperture is close to the Debye length or electric double layer thickness.^11–13^ On the one hand, VMSF has significant size exclusion effect and can effectively exclude large particles (*e.g.* cells) and macromolecules (*e.g.* proteins), showing outstanding anti-fouling ability. On the other hand, the surface of VMSF is negatively charged under conventional pH conditions because of the ionization of Si–OH groups (p*K*_a_ 2 ∼ 3). Thus, VMSF displays remarkable enrichment towards positively charged molecules (especially larger organic cations), producing significant signal amplification. At the same time, electrostatic repulsion to negatively charged molecules leads to good anti-interference properties. Therefore, VMSF shows great potential in modifying electrode to fabricate sensors for direct detection of complex samples.

Until now, VMSF can be conveniently prepared using Stöber solution growth or electrochemically assisted self-assembly (EASA) method.^4,14,15^ The supporting electrode to equip VMSF are mainly divided into two categories. One is indium tin oxide (ITO) electrode, the other is carbon or metal electrode. Owing to covalent bonding between VMSF and ITO surface by forming –Si–O–In or –Si–O–Sn bonds, VMSF can be stably equipped on ITO substrate and exhibits high mechanical stability. However, indium is a rare element with low storage in nature and ITO is of high price. Though carbon or metal electrodes have the advantages of diverse forms (*e.g.*, glassy carbon, graphene, graphite, carbon fibre, gold, platinum, *etc.*) and low detection overpotential, VMSF on these materials is easy to fall off because of weak adhesion. To improve the stability of VMSF, molecular glue (*e.g.*, 3-aminopropyltrimethoxysilane, APTES) or nanomaterial adhesive layer is essential, resulting in complex operation process.^16–18^ Facile and stable equipment of VMSF on efficient and cheap electrode is highly desirable to further explore the applications of VMSF-based sensors.

Electrochemiluminescence (ECL) is a luminescence caused by electron transfer and chemical reaction of reactants at the electrode surface under the excitation of electrochemical process.^19–25^ Compared with other luminescence methods (*e.g.*, fluorescence and chemiluminescence), ECL technique possesses the advantages of zero background and high signal-to-noise, facilitating highly sensitive detection. At the same time, ECL reaction is strictly controlled by electrochemical process and occurs in the diffusion layer near the electrode surface, providing controllable reaction and high sensitivity. Therefore, ECL has been a powerful analysis tool and widely applied in a wide range of applications (*e.g.*, immuno-assay, drug analysis, *etc.*). Among various ECL luminophores, tris(2,2-bipyridine) ruthenium(ii) (Ru(bpy)_3_^2+^) is widely used because of its high ECL efficiency.^26–29^ However, its high price increases the cost of the corresponding analysis. Since Ru(bpy)_3_^2+^ is a large organic cation, the negatively charged VMSF nanochannels can effectively enrich Ru(bpy)_3_^2+^, which significantly increases the concentration of ECL luminophores on electrode surface, leading to high detection sensitivity and low usage of Ru(bpy)_3_^2+^.^12,13^

**Fig. 1:**
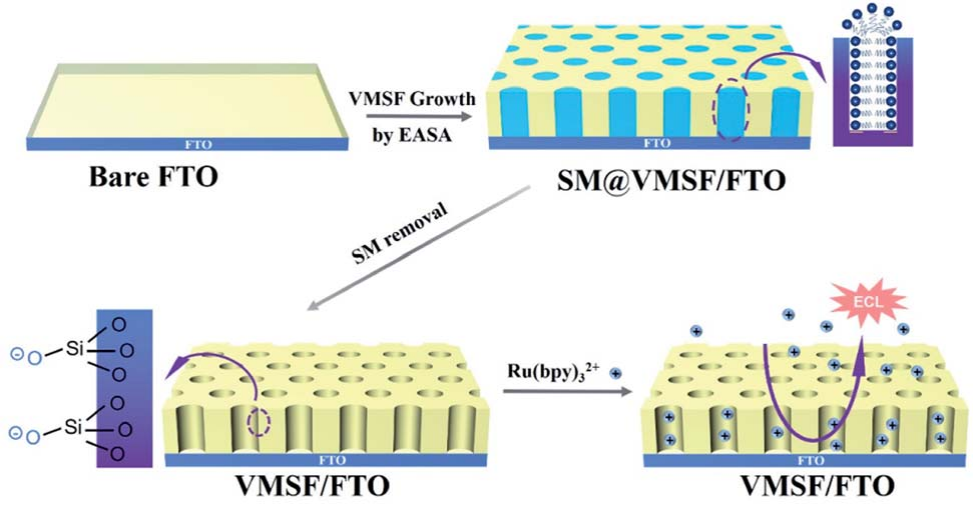
Illustration for the preparation of VMSF/FTO using EASA method and the enrichment of Ru(bpy)_3_^2+^ on negatively charged nanochannels.

In this work, we demonstrate facile equipment of vertically-ordered mesoporous silica-nanochannel film on cheap fluorine-doped tin oxide (FTO) electrodes, which enable sensitive and direct ECL detection of lidocaine in serum. As shown in Fig. 1, VMSF is easily and rapidly grown on the surface of FTO electrode (VMSF/FTO) by EASA method using SM as template. After SM removal, the open nanochannels with negative charge are able to enrich Ru(bpy)_3_^2+^ through the strong electrostatic attraction. Even in absence of co-reactant, the ECL intensity of Ru(bpy)_3_^2+^ can be enhanced nearly 10 times. As a proof-of-demonstration, sensitive and rapid ECL analysis of lidocaine in real serum samples is realized by VMSF/FTO in combination of the antifouling ability of VMSF.

## 2. Experimental

### 2.1 Materials and reagents

Tris(2,2-bipyridine)dichlororuthenium(ii) hexahydrate (Ru(bpy)_3_Cl_2_·6H_2_O) was purchased from Sigma–Aldrich (USA). Tetraethoxysilane (TEOS), lidocaine, cetyltrimethylammonium bromide (CTAB), potassium ferricyanide (K_3_[Fe(CN)_6_]), NaH_2_PO_4_, Na_2_HPO_4_ were obtained from Aladdin (China). The phosphate buffer solution (PBS, 0.01 M, pH 7.0) was prepared by mixing NaH_2_PO_4_ and Na_2_HPO_4_. FTO glass (<10 Ω per square) was obtained from Zhuhai Kaiwo Optoelectronics (China). Human blood serum was obtained by Hangzhou Institute of Occupational Diseases (Hangzhou, China). All other chemicals are of analytical grade and used without further treatment. Ultrapure water (18.2 MΩ cm) was used to prepare all aqueous solutions.

### 2.2 Characterizations

The size, length, and uniformity of VMSF nanochannels were characterized by transmission electron microscopy (TEM). Before characterization, VMSF was gently stripped from the electrode surface and sonicated in ethanol for 5 min. The dispersed sample was dropped on the supporting copper mesh to obtain top-view and cross-sectional TEM images of VMSF on JEM-2100 microscope (JEOL Ltd., Japan). The applied acceleration voltage is 200 kV. Electrochemical measurements including cyclic voltammetry and electrochemical impedance spectroscopy were carried out on PGSTAT302N electrochemical workstation (AutoLab, Switzerland). The ECL measurement was performed on MPI-E II analysis system (Xi'an remex Analytical Instrument Co., Ltd., China).

### 2.3 Preparation of VMSF/FTO electrode

As reported, VMSF was grown on FTO by EASA method.^4,29^ A three-electrode system was used with bare FTO as the working electrode and platinum sheet (Pt, 1 cm × 1 cm) as the counter electrode. Ag/AgCl electrode (saturated KCl solution) was applied as the reference electrode. Before use, FTO (0.5 cm × 1 cm) was firstly washed with NaOH aqueous solution (1 M) and then successively sonicated in acetone, ethanol, and ultrapure water, respectively. To grow VMSF, the precursor solution was prepared with TEOS (13.6 mmol), CTAB (4.35 mmol) in the mixture of NaNO_3_ (0.1 M, 20 mL) and ethanol (20 mL). After pH was adjusted to 3.0 with HCl solution (6 M), the solution was stirred at room temperature for 2.5 h for pre-hydrolysis. Then, bare FTO was immersed in the precursor solution and applied a constant current of −350 μA for 10 s. After the growth of VMSF, the obtained electrode with surfactant micellar (SM) inside the nanochannels (SM@VMSF/ITO) was quickly taken out and thoroughly washed with ultrapure water. After drying with nitrogen (N_2_), SM@VMSF/ITO electrode was aged at 120 °C overnight followed with SM removal by stirring in 0.1 M HCl/ethanol for 5 min. The obtained electrode with open nanochannels was termed as VMSF/FTO.

### 2.4 ECL detection of lidocaine

For ECL detection, PBS (0.1 M, pH 7.0) containing Ru(bpy)_3_^2+^ (10 μM) was used as the supporting solution. To detect lidocaine, VMSF/FTO was immersed in the supporting solution to enrich Ru(bpy)_3_^2+^ for 5 min with stirring. Then, different concentrations of lidocaine were added and the corresponding ECL signals were recorded during cyclic voltammetric scanning. The potential range of cyclic voltammetric scan is 0–1.25 V with a scan rate of 100 mV s^−1^. The voltage of photomultiplier tube in ECL detection was set as 600 V. Standard addition recovery method was applied to detection lidocaine in serum. Specifically, serum with different concentrations of lidocaine was added into the supporting solution and then was detected using the same procedure.

## 3. Results and discussion

### 3.1 Preparation and characterization of VMSF/FTO electrode

As an attractive mesoporous thin film, VMSF can be grown through Stöber solution growth, EASA method, two phase growth method, and magnetically induced orientation, *etc.*^14,30^ Amongst, the EASA method realizes convenient growth of VMSF in a few seconds at room temperature. As illustrated in Fig. 1, VMSF/FTO electrode is prepared by EASA method. Cationic CTAB is applied as the surfactant template and TEOS is the silica precursor. The growth mechanism lies in kinetically controlled interface nucleation/growth by a local pH increase on the surface of electrode generated from the reduction of protons and water molecules under cathodic potentials. This electrochemistry-induced pH gradient facilitates the self-assembly of CTAB semi-micelles on FTO surface as well as the polycondensation of TEOS around the surfactant micelle (SM). After VMSF growth, the VMSF modified electrode filled with SM in the nanochannel (SM/VMSF/FTO) is obtained. After destroying and removing SM with HCl/ethanol, VMSF/FTO with open nanochannels is obtained, which offers potential for electrostatic enrichment of positively charged molecules owing to negatively charged nanochannels from the ionization of Si–OH groups.

**Fig. 2:**
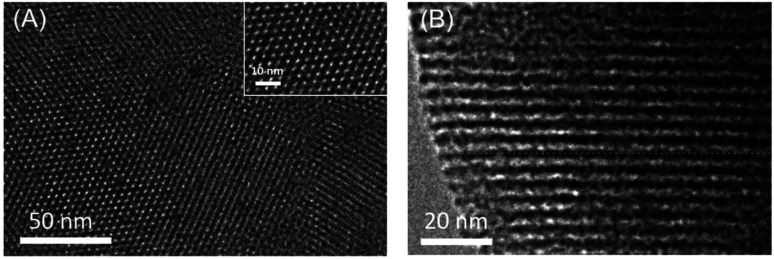
(A) Top-view and (B) cross-sectional TEM images of VMSF. Inset in (A) is TEM image at high magnification.

Fig. 2 illustrates the top-view and cross-sectional TEM images of VMSF stripped from the electrode surface. As shown in the top-view image (Fig. 2A), VMSF has uniformly distributed nanopore structure with no cracks in the observed submicron range. The corresponding TEM image with high magnification (inset in Fig. 2A) reveals highly ordered arrange of nanopore (bright spots in the image) with a diameter of 2–3 nm. Nanochannels with thickness of ∼100 nm is easily identified from the cross-sectional TEM image (Fig. 2B). Thus, VMSF displays ultrasmall and high density nanochannels perpendicular to FTO electrode, which possesses unique advantages including high surface area and fast mass transfer.

**Fig. 3:**
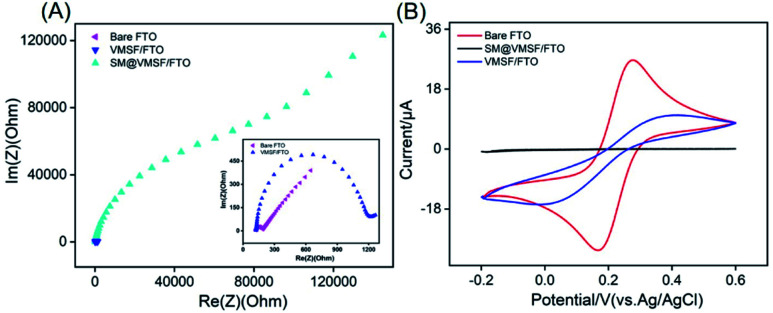
EIS (A) and cyclic voltammetric (B) curves of bare FTO, SM@VMSF/FTO and VMSF/FTO in 0.1 M KCl solution containing 2.5 mM Fe(CN)_6_^3−/4−^. The inset in (A) is the magnified EIS plots of FTO and VMSF/FTO. The scan rate in (B) is 50 mV s^−1^.

The electrochemical behaviour of VMSF/FTO electrode was investigated by electrochemical impedance spectroscopy and cyclic voltammetry using Fe(CN)_6_^3−/4−^ as electrochemical probe. Bare FTO and SM/VMSF/FTO electrodes were also studied for comparison. As shown in Fig. 3A, bare FTO electrode has a very small electron transfer resistance (*R*_ct_), indicating good electron transfer properties. When VSMF is grown and SM is contained in the nanochannel, the obtained SM/VMSF/FTO has significantly increased *R*_ct_, which is more than 400 times larger than that of bare FTO. Obviously, SM in the nanochannel totally hinders the diffusion of the electrochemical probe from the solution to the supporting electrode. When SM is removed, VMSF/FTO with open nanochannel displays slightly higher *R*_ct_ compared with bare FTO. This may be attributed to the electrostatic repulsion of negative VMSF nanochannels towards negative redox probes. The hindered probe diffusion by SM can also be verified through cyclic voltammetric curve (Fig. 3B). In comparison with bare FTO, almost no redox signal is observed on SM/VMSF/FTO, indicating an intact VMSF without crack. Despite permeable nanochannels, VMSF/FTO still exhibits lower signals compared with bare FTO, further proving the electrostatic repulsion of nanochannels to electrochemical probes. In addition, the redox peak of Fe(CN)_6_^3−/4−^ on VMSF/FTO electrode is almost unchanged when the electrode is stored in the refrigerator for one month, indicating stable VMSF modification on FTO. This may be attributed to the formation of Si–O–Sn bonds between VMSF and FTO.

### 3.2 Enhanced ECL of Ru(bpy)_3_^2+^ on VMSF/FTO electrode

**Fig. 4:**
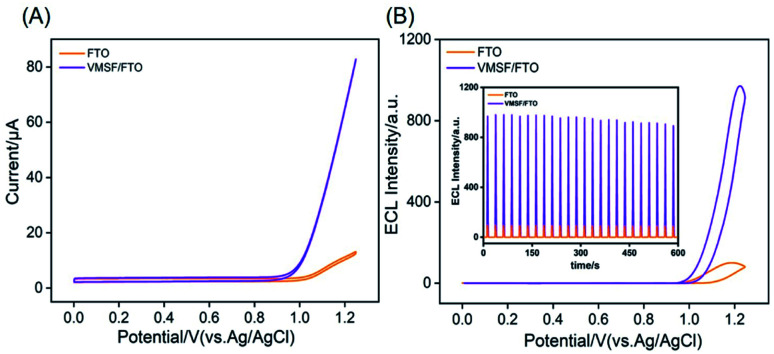
Cyclic voltammetric curves (A) and ECL curves (B) obtained at bare FTO or VMSF/FTO in 0.1 M PBS (pH 7) containing 10 μM Ru(bpy)_3_^2+^. Inset in (B) is the ECL intensity during continuous cyclic voltammetric scan.

VMSF is expected to significantly enrich positively charged molecules (especially large organic cations) due to its high specific surface area and negative charge of nanochannels. As a proof-of-demonstrations, the most commonly used ECL probe, Ru(bpy)_3_^2+^ is chosen as the signal indicator because of its high ECL efficiency and positively charged characteristics. As revealed in Fig. 4A, the VMSF/FTO electrode exhibits significantly large anodic current signal at potentials higher than ∼0.9 V as compared with bare FTO electrode. At the same time, the corresponding ECL intensity of Ru(bpy)_3_^2+^ is ∼10 times higher than that on bare FTO, suggesting VMSF/FTO can significantly enhance the ECL signal of Ru(bpy)_3_^2+^. This is mainly ascribed to the high-efficient enrichment of Ru(bpy)_3_^2+^ by the nanochannels, which increases its microregion concentration on the electrode surface, resulting in amplification of ECL intensity. As depicted in inset of Fig. 4A, the ECL intensity under continuous cyclic voltammetric scans shows a low relative standard deviation (RSD, 3.4%), indicating good stability.

**Fig. 5:**
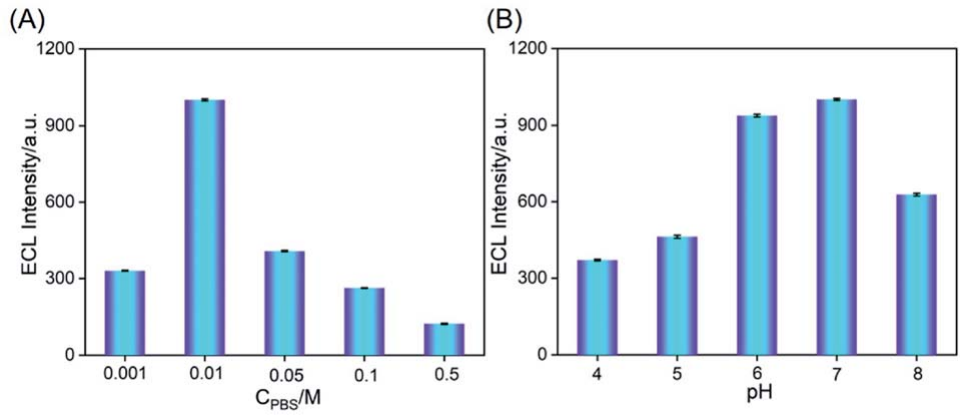
ECL intensity obtained on VMSF/FTO in different concentrations (A) or pH (B) of PBS containing 10 μM Ru(bpy)_3_^2+^.

The charge characteristics and molecular permeability of VMSF nanochannels are significantly affected by ionic strength and pH of the supporting PBS. To obtain the highest enrichment effect of Ru(bpy)_3_^2+^, ionic strength and pH of the supporting PBS are investigated. A neutral PBS (pH 7.0) with different ionic strengths is used. As shown in Fig. 5A, ECL intensity of Ru(bpy)_3_^2+^ in 0.01 M PBS is the highest. At low ionic strength (the buffer concentration is less than 0.1 M), the electrical double layer (EDL) trends to overlap in the ultrasmall space of nanochannel (∼2 nm in diameter). In this case, VMSF nanochannels are forced to attract counter ions to decay electrostatic surface potential, leading to permselectivity of nanochannels with selective permeability of cationic ions and repelling of anions.^31^ In other words, low ionic strength facilitates the enrichment of Ru(bpy)_3_^2+^. As 0.001 M PBS might lead to low conductivity, 0.01 M PBS with the highest ECL signal is chosen for further investigation. ECL signals at different pH are shown in Fig. 5B. When pH increases from 4 to 7, the ECL strength of Ru(bpy)_3_^2+^ on VMSF/FTO electrode increases, resulting from the increase of negative charge density of nanochannels due to the increased ionization of silicon hydroxyl at high pH. The weak decrease of ECL signal at pH 8 may be attributed to the decreased stability of silica under alkaline conditions. Thus, pH 7 is applied in further detection.

### 3.3 Sensitive ECL detection of lidocaine

Lidocaine (2-diethylamino-*N*-(2,6-dimethylphenyl)acetamide, abbreviated as Lid), as an anesthetic and antiarrhythmic drug, has obvious biphasic effects of excitation and inhibition on the central nervous system. When the blood concentration is low, analgesia, drowsiness and pain threshold increase, while the effect or toxicity increased with the increase of dose and the sub-toxic concentration had anticonvulsant effect.^26^ Thus, sensitive detection of lidocaine in blood is of great significance. In comparison with the developed techniques for the detection of lidocaine including electrochemistry, fluorescence, and chemiluminescence, ECL sensing has great potential for sensitive detection due to the advantages of low background, high signal-to-noise, and wide dynamic range.

**Fig. 6:**
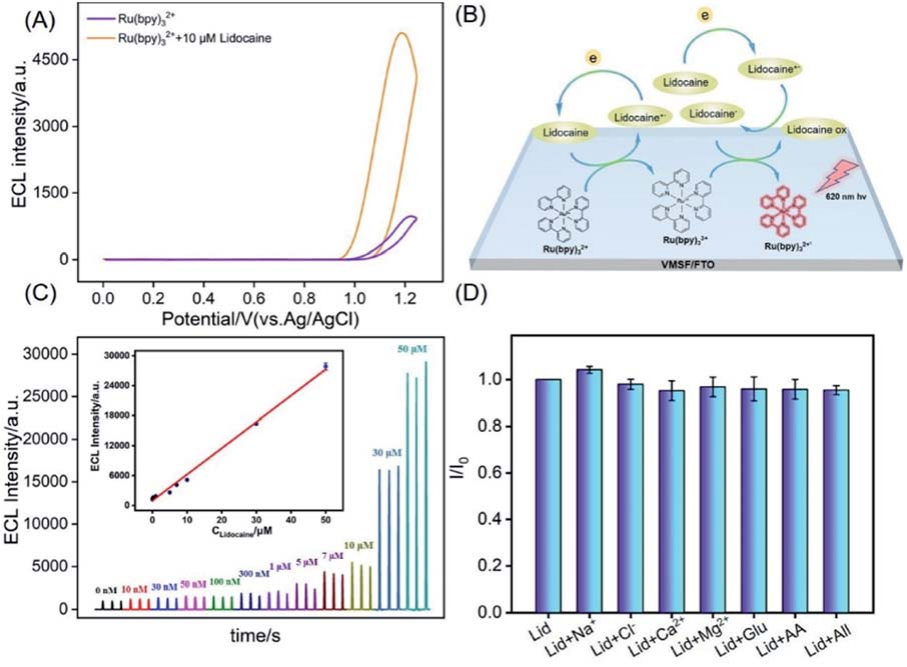
(A) Cyclic voltammetric curves obtained at VMSF/FTO in Ru(bpy)_3_^2+^ solution (10 μM in 0.1 M PBS, pH 7) in absence or presence of lidocaine. (B) The illustration for the possible ECL mechanism. (C) ECL intensities with different concentrations of lidocaine. The inset is calibration curve for lidocaine determination. (D) The ECL intensities ratio (*I*/*I*_0_) obtained on VMSF/FTO for detection of 20 μM lidocaine (Lid) in the absence (*I*) and presence (*I*_0_) of added interfering species (1 mM). The concentration of the interfering species is 1 mM.

As revealed in Fig. 6A, significant ECL increase of Ru(bpy)_3_^2+^ is observed in presence of lidocaine, indicating that lidocaine can act as the co-reactant to promote the ECL of Ru(bpy)_3_^2+^. A possible mechanism of the ECL enhancement is shown in Fig. 6B. At high potential, Ru(bpy)_3_^2+^ is oxidized to Ru(bpy)_3_^3+^. At the same time, lidocaine is oxidized to its cationic free radicals, which produces a high-energy intermediate with strong reduction ability through deprotonation. Then, this intermediate reduces Ru(bpy)_3_^3+^ to form the excited state [Ru(bpy)_3_^2+^]*, which produces ECL when returns to the ground state.

Fig. 6C shows the ECL signals obtained on VMSF/FTO electrode with different concentrations of lidocaine in PBS containing Ru(bpy)_3_^2+^ (10 μM). A good linear correlation is found between the ECL intensity (*I*_ECL_) and the concentration of lidocaine (*C*_lidocaine_) from 10 nM to 50 μM (*I*_ECL_ = 525 *C*_lidocaine_ + 983, *R*^2^ = 0.995). The limit of detection (LOD) is determined as low as 8 nM. Lidocaine is usually synthesized by the reaction between *N*-(2,6-dimethylphenyl)chloroacetamide and diethylamine with 70–85% yield.^32^ These two raw compounds might coexist in lidocaine. However, *N*-(2,6-dimethylphenyl)chloroacetamide cannot be used as a co-reactant of Ru(bpy)_3_^2+^. In case of diethylamine, it can be used as a co-reactant of Ru(bpy)_3_^2+^ due to the presence of secondary amine. However, the enhancement effect of ethylenediamine on ECL signal is not significant compared with compound with tertiary amine structure. Combining high purity and low linear detection range of lidocaine, the impurity of lidocaine will not affect the detection performance. Comparison between detection of lidocaine using different methods is given in Table S1 (ESI†).^33–38^ The LOD is lower than that obtained by an ECL sensor based on luminol-doped silica nanoparticles,^33^ or by capillary electrophoresis (CE),^34^ fluorescent sensor,^35^ high-performance liquid chromatography (HPLC),^36,37^ but slightly higher than that obtained using an ECL sensor based on 3D graphene paper electrode (GPE).^38^

### 3.4 Detection selectivity and real sample analysis

To detect lidocaine in serum, the selectivity of the detection is also evaluated using possible interference that exist in serum matrix. The tested interferences include ions (Na^+^, Ca^2+^, Cl^−^, Mg^2+^), and biological small molecule such as glucose (Glu) or ascorbic acid (AA). As shown in Fig. 6D, one or the mixture of the above substances will not interfere with the detection of lidocaine, indicating high selectivity. The detection of lidocaine in serum is performed using standard addition method. As shown in Table 1, serum samples with artificially spiked lidocaine are examined with satisfactory recoveries ranged from 95.0% to 107.0%. Thus, the developed sensor displays good reliability owing to the integration with VMSF, which offers high anti-fouling characteristics. The RSD for three measurements is less than 3.3%, indicating high reproducibility.

**Table 1:** ECL determination of lidocaine in serum

Sample	Added (μM)	Found (μM)	RSD (%, *n* = 3)	Recovery (%)
Serum	1.00	1.04	3.3	104.0
	5.00	5.33	1.1	106.6
	15.00	15.18	1.4	101.2

## 4. Conclusions

In summary, we have developed a sensitive ECL sensor based on facile equipment of VMSF on FTO electrode, which enable rapid and convenient detection of lidocaine in serum. VMSF is easily grown on the surface of FTO at room temperature in a few seconds and exhibits high stability. Owing to significant enrichment of Ru(bpy)_3_^2+^ by nanochannels with negative charges, the developed VMSF/FTO electrode displays ∼10 times enhanced ECL signal even without co-reactants. As lidocaine can act as co-reactor and remarkably increase the ECL of Ru(bpy)_3_^2+^, sensitive and direct detection of lidocaine in serum is realized by the sensor in combination with the anti-fouling ability of VMSF. It has been established that molecules with tertiary amine can act as efficient co-reactant of Ru(bpy)_3_^2+^. The developed sensor would also be sensitive enough for other active compounds with tertiary amine similar to lidocaine taking advantages of efficient enrichment of Ru(bpy)_3_^2+^ and detection using co-reactant mechanism. Owing to the easy fabrication, cheap electrode, and significantly enhanced ECL, the developed VMSF/FTO sensor may be extended when the nanochannels are further functionalized.

## Author contributions

Renchuan Liang: data curation, Jinghang Jiang: data curation, Yanyan Zheng: data curation, Ajabkhan Sailjoi: data curation, Jie Chen: writing-reviewing and editing, Jiyang Liu: supervision, writing-reviewing and editing, Hongxue Li: supervision, writing-original draft preparation.

## Conflicts of interest

There are no conflicts to declare.

## Supplementary Material

RA-011-D1RA06375H-s001
