# Supplementary material for: Vertically oriented mesoporous silica film modified fluorine-doped tin oxide electrode for enhanced electrochemiluminescence detection of lidocaine in serum
Source: RSC Adv. 2021 Oct 26;11(55):34669–75. doi: 10.1039/d1ra06375h (PMC9042675; doi:10.1039/d1ra06375h)
Supplement: RA-011-D1RA06375H-s001 [file RA-011-D1RA06375H-s001.pdf]

Electronic Supplementary Material (ESI) for RSC Advances.
This journal is © The Royal Society of Chemistry 2021

Table S1 Comparison between detection of lidocaine using different methods.

**Material**	**Method**	**Linear range (μM)**	**Detection limit (nM)**	**References**
luminol-SiNPs/GCE	ECL	0.5-100	92.5	33
SA-DLLME-FASS	CE	0.05–1	10	34
N-CDs	Fluorescent	185.0-1295.0	54000	35
HF-LLLME	HPLC	0.2-8.5	42.6	36
Fe_3O_4@SiO_2-C_{18} nanoparticles	HPLC	0.2-10.6	42.6	37
3D GPE	ECL	0.01-10<br>50-10000	7.8	38
VMSF/FTO	ECL	0.01-50	8	our work

luminol-SiNPs, luminol-doped silica nanoparticles; GCE, glassy carbon electrode; SA-DLLME-FASS, Surfactant-assisted-dispersive liquid-liquid microextraction-field-amplified sample stacking; CE, capillary electrophoresis; N-CDs, N doped carbon dots; HF-LLLME, hollow fiber liquid-liquid-liquid microextraction; HPLC, high-performance liquid chromatography; Fe_3O_4@SiO_2-C_{18}, C18-functionalized magnetic silica nanoparticles; 3D GPE, 3D graphene paper electrode.
